# Safety and efficacy of tofacitinib in children with ulcerative colitis complicated with arthropathy: a single-center study

**DOI:** 10.3389/fped.2025.1643668

**Published:** 2025-11-14

**Authors:** Shijian Miao, Shengnan Wang, Wenhui Hu, Ying Huang, Yu Shi

**Affiliations:** 1 Department of Gastroenterology, National Children’s Medical Center, Children’s Hospital of Fudan University, Shanghai, China; 2 Department of Rheumatology, National Children’s Medical Center, Children’s Hospital of Fudan University, Shanghai, China

**Keywords:** arthropathy, tofacitinib, ulcerative colitis, children, safety and efficacy

## Abstract

**Objectives:**

Tofacitinib is an oral Janus kinase (JAK) inhibitor initially used for the treatment of arthritis. It demonstrated to effectively induce and maintain remission in adults with inflammatory bowel disease (IBD). However, data on its safety and efficacy in children with ulcerative colitis (UC), particularly in children with comorbid arthropathy, remained limited. This study aimed to evaluate the safety and efficacy of tofacitinib in treating children with UC who also had comorbid arthropathy.

**Methods:**

We conducted a retrospective cohort study enrolling children with UC and comorbid arthropathy who received tofacitinib treatment at the Gastroenterology Department of the Children's Hospital of Fudan University from January 2018 to December 2024. All enrolled UC patients underwent blood tests, stool tests, and colonoscopies, with the Pediatric Ulcerative Colitis Activity Index (PUCAI) used to assess clinical indicators, clinical response, and clinical remission.

**Results:**

A total of 16 patients met the inclusion criteria, all of whom presented with comorbid arthropathy. The mean age at onset was 7.1 ± 3.7 years, with a mean body mass index (BMI) of 14.6 ± 2.0 kg/m^2^. All patients had previously failed biologic therapy with infliximab. The majority patients initiated tofacitinib treatment at a starting dose of 2.5 mg twice daily (bid) and adjusted based on clinical response, with a maximum dose of 5 mg bid. Fecal calprotectin and endoscopic scores decreased significantly by weeks 14, 21, and 30, while albumin and BMI levels increased (all *p* < 0.05). The mean PUCAI scores also demonstrated a significant decline. One patient (6.25%) achieved clinical response by week 7, nine (56.25%) by week 14, and five (31.25%) by week 21. Six patients (37.5%) achieved clinical remission by week 30.

**Conclusions:**

Our study provided promising evidence for the safety and efficacy of tofacitinib as part of the treatment regimen for children with UC complicated with arthropathy. Further large-scale, prospective studies are needed to confirm these findings.

## Introduction

Inflammatory bowel diseases (IBD), including Crohn disease (CD) and ulcerative colitis (UC), are chronic, relapsing inflammatory conditions of the gastrointestinal tract. The prevalence of IBD is increasing globally, particularly among children (1). Symptoms include weight loss, diarrhea, bloody stools, and abdominal pain. Currently, there is no cure for IBD, and the primary treatment goal is to maintain clinical remission without the use of glucocorticoids (2). Treatment options include conventional pharmacological therapies and surgical intervention (3). Beyond gastrointestinal symptoms, up to 40% of children with IBD experience extraintestinal manifestations (EIMs), which can involve the joints, skin, eyes, and hepatobiliary system. These EIMs contribute significantly to disease burden, with studies reporting that children with EIMs have poorer health-related quality of life (QoL) compared to those without, including increased pain, fatigue, and psychosocial distress. For instance, musculoskeletal EIMs—such as arthritis—affect 10%–20% of childhood IBD cases and are a leading cause of disability, while dermatologic and ocular manifestations further exacerbate morbidity. Given their profound impact on physical and emotional well-being, early recognition and multidisciplinary management of EIMs are essential to improving outcomes in children with IBD (4–6). In some cases, arthritis may precede gastrointestinal symptoms (7, 8).

Targeting the Janus kinase (JAK) pathway has emerged as a promising therapeutic strategy for IBD. Tofacitinib, an oral JAK inhibitor, selectively inhibits JAK1 and JAK3 in the JAK-STAT pathway, thereby reducing proinflammatory cytokines (4). Approved by the US Food and Drug Administration (FDA) in 2012 for severe active rheumatoid arthritis and in 2018 for UC, tofacitinib has been available in China for the past two years. However, data on its use in chilldhood IBD, particularly in patients with associated inflammatory arthropathy, remain limited (5). Therefore, we conducted a retrospective cohort study to evaluate the efficacy and safety of tofacitinib in children with UCcomplicated by arthropathy.

## Methods

### Study cohort

**Figure 1:**
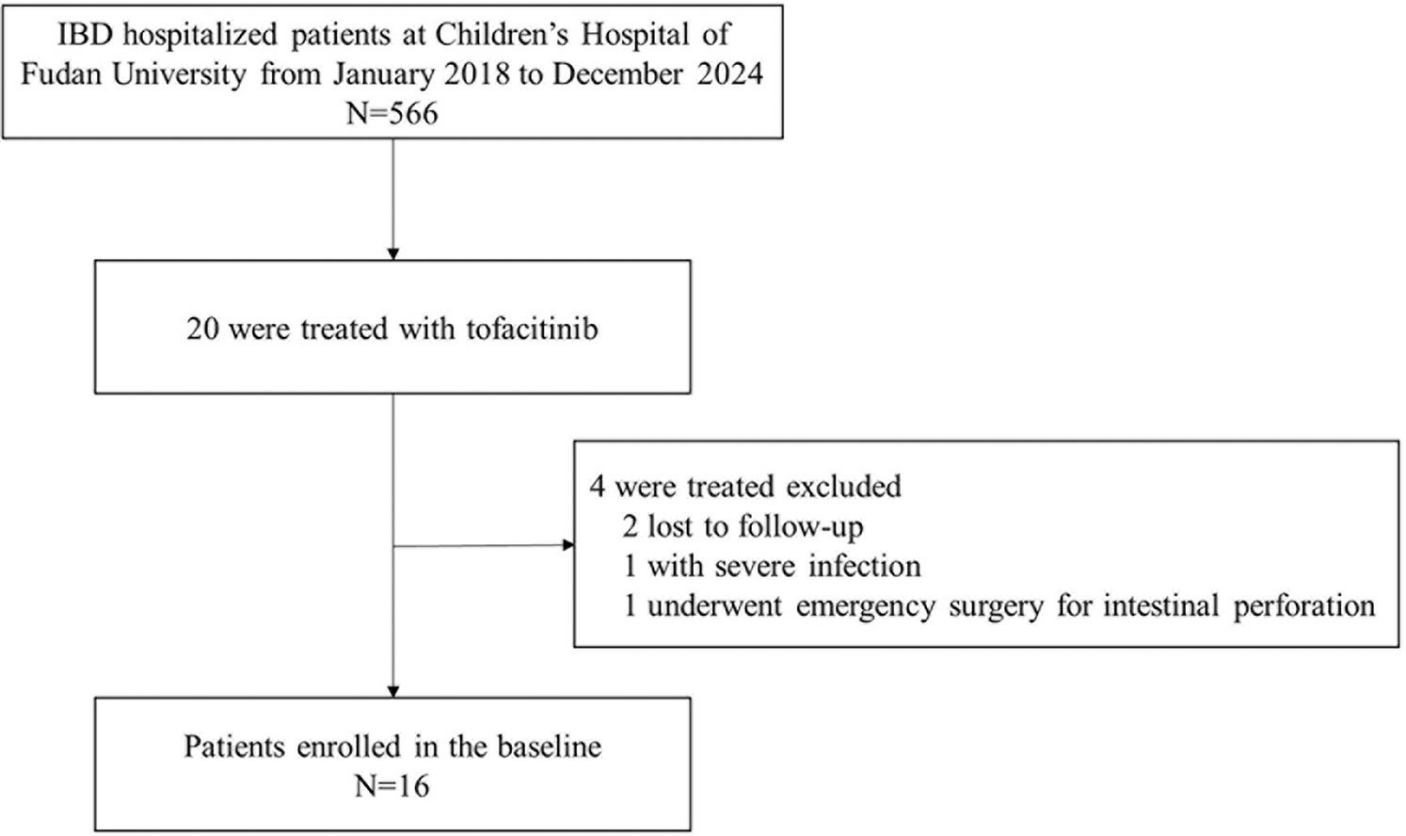
Patient inclusion flowchart.

We conducted a perspective cohort study at the IBD Center of the Children's Hospital of Fudan University from January 2018 to December 2024 (Figure 1).

### Inclusion criteria

1. Patients must meet the clinical, laboratory, and endoscopic diagnostic criteria outlined in Inflammatory Bowel Disease in Children and Adolescents: Recommendations for Diagnosis–The Porto Criteria (2005).
2. Patients were required to have arthritis manifestations (either monoarticular or polyarticular) confirmed by imaging studies.
3. Tofacitinib must be administered for the first time.

### Exclusion criteria

1. Patients with incomplete clinical data.
2. Patients requiring urgent surgical intervention due to complications such as intestinal obstruction, perforation, or gastrointestinal bleeding.
3. Patients with severe underlying conditions, including malignancies, moderate-to-severe heart failure, or multiple organ dysfunction syndrome.
4. Patients with active infections such as tuberculosis or hepatitis B virus infection.
5. Patients with thrombotic disorders or coagulation dysfunction.

### Data collection

We reviewed electronic medical records to collect demographic, clinical, laboratory, radiographic, and endoscopic data. Baseline characteristics included patient demographics (age, gender, BMI, medical history), disease features (symptoms, UC subtype), tofacitinib treatment details (induction and maintenance doses, duration, prior therapies), biochemical markers (serum C-reactive protein, albumin, hemoglobin), fecal calprotectin and endoscopic findings. Clinical response and adverse events were assessed at weeks 7, 14, 21, and 30.

### Clinical outcomes

We evaluated PUCAI scores at weeks 7, 14, and 21. Disease activity was categorized as follows: 0–9 (no activity), 10–34 (mild activity), 35–64 (moderate activity), and 65–85 (severe activity) (6). Adverse events previously associated with tofacitinib were retrospectively assessed. Treatment failure was defined as the absence of significant symptom improvement, discontinuation of tofacitinib, or referral for surgery. Relapse was defined as therapeutic failure after an initial response or discontinuation of treatment.

Clinical remission was defined as a PUCAI score < 10 and corticosteroid-free treatment without the need for new medications or colectomy. Clinical response was defined as a decrease in PUCAI score by ≥20 or by one activity level from baseline.

### Statistical analysis

Data were analyzed using SPSS 26.0. Continuous variables were expressed as mean ± standard deviation (SD) or median (interquartile range), while categorical data were expressed as percentages and absolute numbers. A *P*-value <0.05 was considered statistically significant.

## Results

### General and clinical characteristics

Sixteen patients were included in the study. The male-to-female ratio was approximately 3:5. The mean age at onset was 7.1 ± 3.7 years, with a mean weight of 18.7 ± 8.0 kg, height of 109.9 ± 20.8 cm, and BMI of 14.6 ± 2.0 kg/m^2^. All patients presented with abdominal pain, diarrhea, hematochezia, and tenesmus. Anemia and hypoalbuminemia were observed in 14 patients (87.5%), while fever was reported in five (31.3%). All 16 children presented with arthropathy, of which 4 cases were peripheral arthritis and 12 cases were axial arthritis. The Jadas 10 score was 12.7 ± 4.1 (7) [1] (Table 1).

**Table 1:** Baseline characteristics in the 16 UC patients.

Baseline demographics	*n* = 16
Male, *n* (%)	6 (37.5%)
Age, year, median	7.1 ± 3.7
Weight, kg, median	18.7 ± 8.0
Height, cm, median	109.9 ± 20.8
BMI, kg/m^2^, median	14.6 ± 2.0
Symptoms, *n* (%)	
Abdominal pain	16 (100)
Hematochezia	16 (100)
Diarrhea	16 (100)
Tenesmus	16 (100)
Anemia	14 (87.5)
Fever	5 (31.3)
Hypoalbuminemia	14 (87.5)
Peripheral arthritis	4 (25)
Axial arthritis	12 (75)
Jadas 10	12.7 ± 4.1
IBD subtype, *n* (%)	
UC	16 (100)
UC characteristics, *n* (%)	
Left-sided colitis	2 (12.5)
Pancolitis	14 (87.5)
Biologic therapy before tofacitinib initiation, months	
Infliximab	12.3 ± 4.2

UC, ulcerative colitis.

### Clinical response and remission

**Figure 2:**
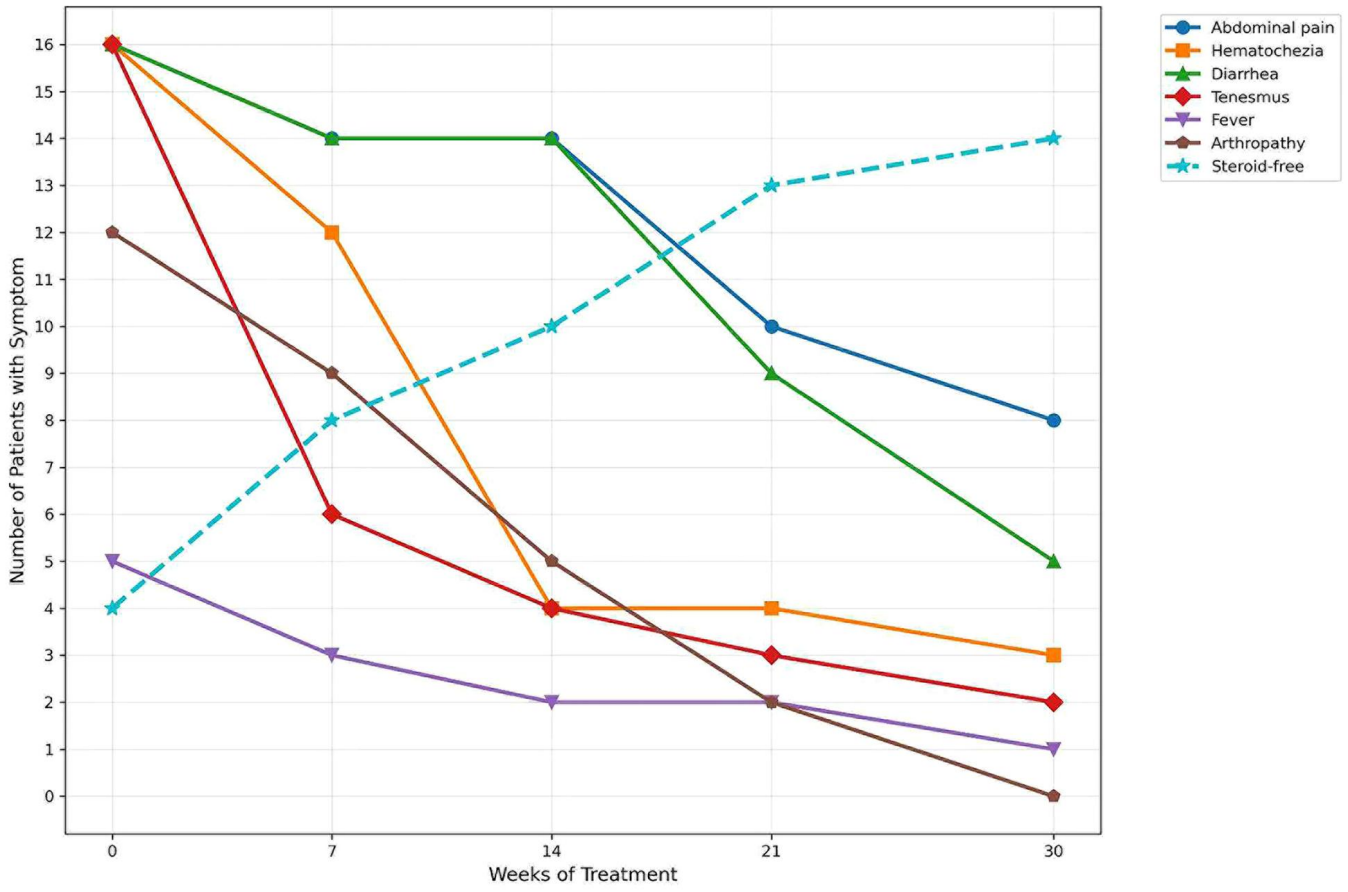
During JAK treatment, patients with IBD experience improvements in their digestive systems and systemic symptoms.

Clinical symptoms, including abdominal pain, hematochezia, diarrhea, tenesmus, and arthropathy, improved significantly by weeks 7, 14, 21, and 30. All patients had previously failed infliximab therapy, with a median washout period of 12.3 ± 4.2 months. Twelve patients (75.0%) were on steroids at the initiation of tofacitinib. By week 30, only 2 patients (87.5%) discontinued steroids (Figure 2).

Fecal calprotectin decreased significantly by weeks 14, 21, and 30, while albumin and BMI levels increased (all *P* < 0.05). Endoscopic scores (Mayo score) showed a significant reduction from weeks 0 to 30. Jadas 10 score decreased from weeks 0 to 30. The mean PUCAI score decreased from 45.0 ± 5.4 to 11.9 ± 6.0. At the 0-week, all sixteen patients were in moderate disease activity. From week 7 to week 30, the number of patients with no active disease increased to six (37.5%), and the number of patients with mild activity increased to ten (62.5%) (Table 2). One patient (6.25%) achieved clinical response by week 7, and nine (56.25%) at week 14, five (31.25%) at week 21. Six patients (37.5%) achieved clinical remission by week 30.

**Table 2:** Laboratory and clinical parameter shifts under JAK inhibitor treatment.

Laboratory indicators	Week 0	Week 7	Week 14	Week 21	Week 30
CRP (mg/L)	19.7 ± 29.6	17.8 ± 33.9	12.7 ± 25.2	4.0 ± 3.6	3.6 ± 5.0
ESR (mm/h)	72.8 ± 34.1	58.9 ± 30.1	52.9 ± 26.4^b^	37.6 ± 20.6^c^^,^^f^	33.4 ± 17.5^d^^,^^g^^,^^i^
WBC (×10^9^/L)	11.4 ± 5.4	11.4 ± 5.0	9.3 ± 3.5	9.1 ± 3.3	9.9 ± 4.5
Plt (×10^9^/L)	442.7 ± 144.6	362.8 ± 131.8	428.6 ± 135.6	382.4 ± 150.7	354.9 ± 89.8
Hb (g/L)	92.8 ± 17.6	98.6 ± 14.3	108.1 ± 18.2^b^	118.0 ± 16.4^c^^,^^f^	118.7 ± 16.9^d^^,^^g^
Albumin (g/L)	35.6 ± 2.8	37.3 ± 3.1	38.6 ± 3.1^b^	39.4 ± 2.5^c^^,^^f^	40.4 ± 2.5^d^^,^^g^
BMI (kg/m^2^)	14.6 ± 2.0	15.1 ± 2.0	15.9 ± 2.2	16.4 ± 2.5^c^	16.9 ± 2.7^d^^,^^g^
Jadas 10	12.7 ± 4.1	9.9 ± 3.8^a^	5.4 ± 2.7^b^^,^^e^	2.9 ± 1.8^c^^,^^f^^,^^h^	6.5 ± 5.1^d^^,^^g^^,^^i^
Fecal calprotectin (μg/g)	461.3 ± 294.6	350.1 ± 208.0	228.6 ± 158.7^b^	180.7 ± 127.7^c^^,^^f^	142.9 ± 134.1^d^^,^^g^
Mayo score	10.6 ± 2.1	–	–	–	4.7 ± 4.2^d^
PUCAI Score	45.0 ± 5.4	37.2 ± 3.9^a^	29.2 ± 6.9^b^^,^^e^	17.8 ± 5.4^c^^,^^f^^,^^h^	11.9 ± 6.0^d^^,^^g^^,^^i^^,^^j^
*n* (%)					
No activity (0–9)	0	0	0	0	6 (37.5%)
Mild activity (10–34)	0	1 (6.25%)	10 (62.5%)	16 (100%)	10 (62.5%)
Moderate activity (35–64)	16 (100%)	15 (93.75%)	6 (37.5%)	0	0
Severe activity (65–85)	0	0	0	0	0

a Week 0 vs. week 7.

b Week 0 vs. week 14.

c Week 0 vs. week 21.

d Week 0 vs. week 30.

e Week 7 vs. week 14.

f Week 7 vs. week 21.

g Week 7 vs. week 30.

h Week 14 vs. week 21.

i Week 14 vs. week 30.

j Week 21 vs. week 30.

### Dose

**Figure 3:**
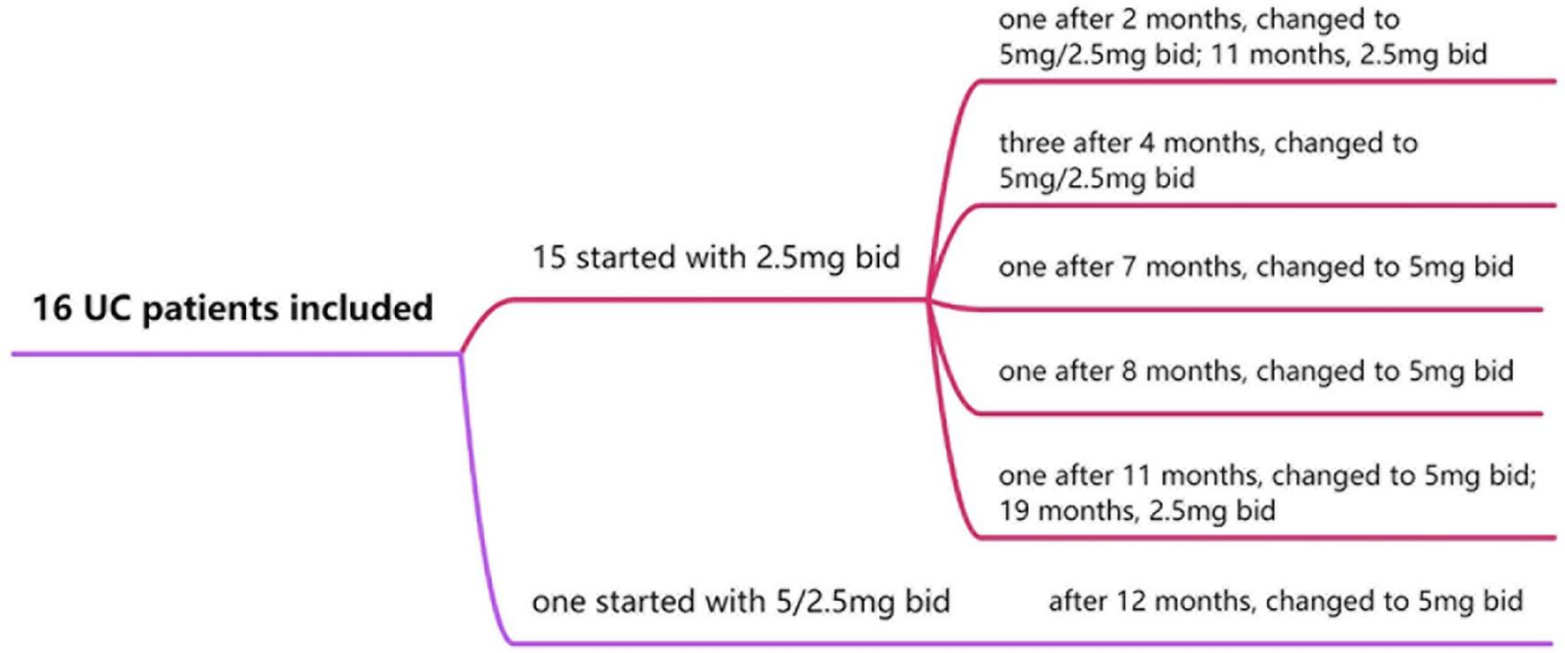
Treatment flow chart of subject treatment included in analysis.

All children with UCcompleted the 30-week treatment course. Tofacitinib dosages were adjusted based on clinical symptoms, joint scores, changes in serum C-reactive protein (CRP) and fecal calprotectin level. Fifteen children started with an initial dose of 2.5 mg twice daily (bid). Among them: 1 case was adjusted to 5 mg in the morning and 2.5 mg in the evening at 2 months, then reduced to 2.5 mg bid at 11 months; 3 cases were adjusted to 5 mg in the morning and 2.5 mg in the evening at 4 months; 2 cases were escalated to 5 mg bid at 7 and 8 months, respectively; 1 case was increased to 5 mg bid at 11 months and later reduced to 2.5 mg bid at 19 months. The remaining 1 patient started at 5 mg in the morning and 2.5 mg in the evening, then adjusted to 5 mg bid at 12 months (Figure 3).

### Adverse events

No serious drug-related adverse events were reported, and no thromboembolic events were observed. Eight mild infectious events were recorded in 16 patients, none of which required discontinuation of tofacitinib.

## Discussion

Our study demonstrated the potential of tofacitinib as a salvage therapy for childhood IBD, particularly in patients with inflammatory arthritis. By week 30, 37.5% of patients achieved corticosteroid-free remission, with significant improvements in clinical symptoms and joint symptoms. Additionally, reductions were observed in fecal calprotectin and endoscopic scores, along with a notable decline in PUCAI score. Adverse events were limited to mild infections.

This study represented the first Asian report on the efficacy and safety of tofacitinib in childhood UC with associated arthropathy. Among the patients, one (6.25%) achieved clinical response by week 7, nine (56.25%) by week 14, and five (31.25%) by week 21. Overall, six patients (37.5%) achieved clinical remission by the 30-week mark. Tofacitinib demonstrated moderate efficacy in both inducing and maintaining remission in children with UC. In the OCTAVE Induction 1 and 2 trails–two Phase III clinical studies—the induction relief effect of tofacitinib (10 mg twice daily) was evaluated after 8 weeks of treatment. The results revealed that the tofacitinib group had significantly higher rates of clinical remission (18.5% vs. 8.2%, *p* < 0.001) and endoscopic improvement (31.3% vs. 15.6%, *p* < 0.001) compared to the placebo group (7). The OCTAVE Sustain study further assessed the efficacy of tofacitinib in maintenance therapy. Patients receiving tofacitinib (5 mg or 10 mg twice daily) exhibited significantly higher clinical remission rates at 52 weeks compared to the placebo group (34.3% vs. 40.6% vs. 11.1%, *p* < 0.001) (8). Recent real-word studies on tofacitinib in adults have reported higher remission rates than those observed in our study. For instance, a multicenter study in the United Kingdom by Honap et al. demonstrated a 74% response rate and a 44% corticosteroid-free remission rate at week 8. However, this cohort was less refractory to prior biologics, with 18% being biologic naïve and only 36% refractory to two biologics. Notably, in this adult study, patients with primary non-response were significantly younger than responders, suggesting that the younger age of our cohort may have influenced response rates (9). Similarly, in a Spanish real-life cohort study by Chaparro et al. (10), 60% of patients achieved a response, and 31% attained clinical remission by week 8.

Tofacitinib has been explored as a treatment option for refractory IBD in several studies. For instance, one study demonstrated that among 19 patients with severe refractory CD, 58% were able to continue tofacitinib treatment, and 46% exhibited a positive response during endoscopic evaluation (11). In another study involving 21 children and adolescents aged 2–18 years, tofacitinib treatment led to a significant reduction in clinical activity index over 52 weeks, with some patients achieving clinical remission (12). Additionally, a retrospective study reported that 71% of children treated with a combination of tofacitinib and biologics achieved a glucocorticoid-free response at the 6-month mark (13).

Tofacitinib has been approved by the US FDA for the treatment of active polyarticular juvenile idiopathic arthritis (JIA) in children aged 2 years and older, particularly for patients who have shown inadequate response or intolerance to one or more TNF inhibitors (14). Clinical trials have demonstrated that tofacitinib significantly reduces the risk of disease flare-ups and improves disease activity levels. For example, in a 48-week Phase III clinical trial, the JIA-ACR70 and JIA-ACR90 response rates for the tofacitinib group were 60.0% and 33.6%, respectively, with efficacy progressively improving over time. Additionally, 26% of patients achieved disease-free status within 44 weeks of treatment (15). Arthropathy is a common complication in patients with IBD, particularly in those with CD and UC. It often presents as peripheral arthropathy, frequently affecting joints such as the hips and knees (16). While tofacitinib is primarily used to manage intestinal inflammation, its mechanism of action—targeting the JAK-STAT signaling pathway—may also help control systemic inflammation associated with IBD, including arthropathy (12). Tofacitinib has shown promise in treating IBD-related arthropathy, especially in cases where conventional therapies have failed (17). At our center, we observed that tofacitinib may indirectly alleviate joint symptoms by improving intestinal inflammation, particularly in patients who did not respond to traditional treatments. These findings align with other studies, highlighting the potential dual benefit of tofacitinib in managing both intestinal and extraintestinal manifestations of IBD. Additionally, tofacitinib is an oral small-molecule drug, which is more cost-effective compared to biologic agents. This makes it a more accessible and affordable treatment option for children in less developed regions.

The safety profile of tofacitinib is consistent with that of other JAK inhibitors, with common adverse events including infections (such as herpes zoster), upper respiratory tract infections, non-melanoma skin cancer, and cardiovascular events (18, 19). In the OCTAVE Sustain trial, the tofacitinib group exhibited higher rates of overall infections and herpes zoster compared to the placebo group, but no significant increase in serious adverse events was observed (18). However, tofacitinib has been associated with an elevated risk of cardiovascular events, such as thrombosis and myocardial infarction (20, 21). In our study, drug-related adverse events were limited to mild infections, with no reported cases of thromboembolic events.

Tofacitinib is typically administered twice daily, with a recommended dose of either 5 mg or 10 mg. Clinical trials have demonstrated that the 10 mg dose offers high efficacy and a favorable safety profile (22, 23). In some studies, tofacitinib dosages have ranged from 0.5 mg to 15 mg, with the 10 mg dose significantly improving clinical and endoscopic response rates by week 8 (24). For children, the dosage may need to be adjusted based on age and weight (25). At our center, treatment is initiated at a dose of 2.5 mg bid and adjusted according to clinical response, with a maximum dose of 5 mg bid.

In conclusion, our study supports the efficacy and safety of tofacitinib in the treating children with UC and associated arthritis. But our study had a relatively small sample size, which may limit the statistical power and generalizability of the results. However, multicenter and long-term studies are still needed to further evaluate its therapeutic effects and safety profile.

## Data Availability

The raw data supporting the conclusions of this article will be made available by the authors, without undue reservation.
